# Supplementary material for: Intrinsic ion dynamics underlies the temporal nature of resting-state functional connectivity
Source: bioRxiv. 2025 Nov 9:2025.11.08.687387. Preprint. [Version 1] doi: 10.1101/2025.11.08.687387 (PMC12637618; doi:10.1101/2025.11.08.687387)
Supplement: 1 [file NIHPP2025.11.08.687387V1-supplement-1.pdf]

bioRxiv preprint doi: https://doi.org/10.1101/2025.11.08.687387; this version posted November 9, 2025. The copyright holder for this preprint (which was not certified by peer review) is the author/funder, who has granted bioRxiv a license to display the preprint in perpetuity. It is made available under aCC-BY 4.0 International license.

Supplementary Figure

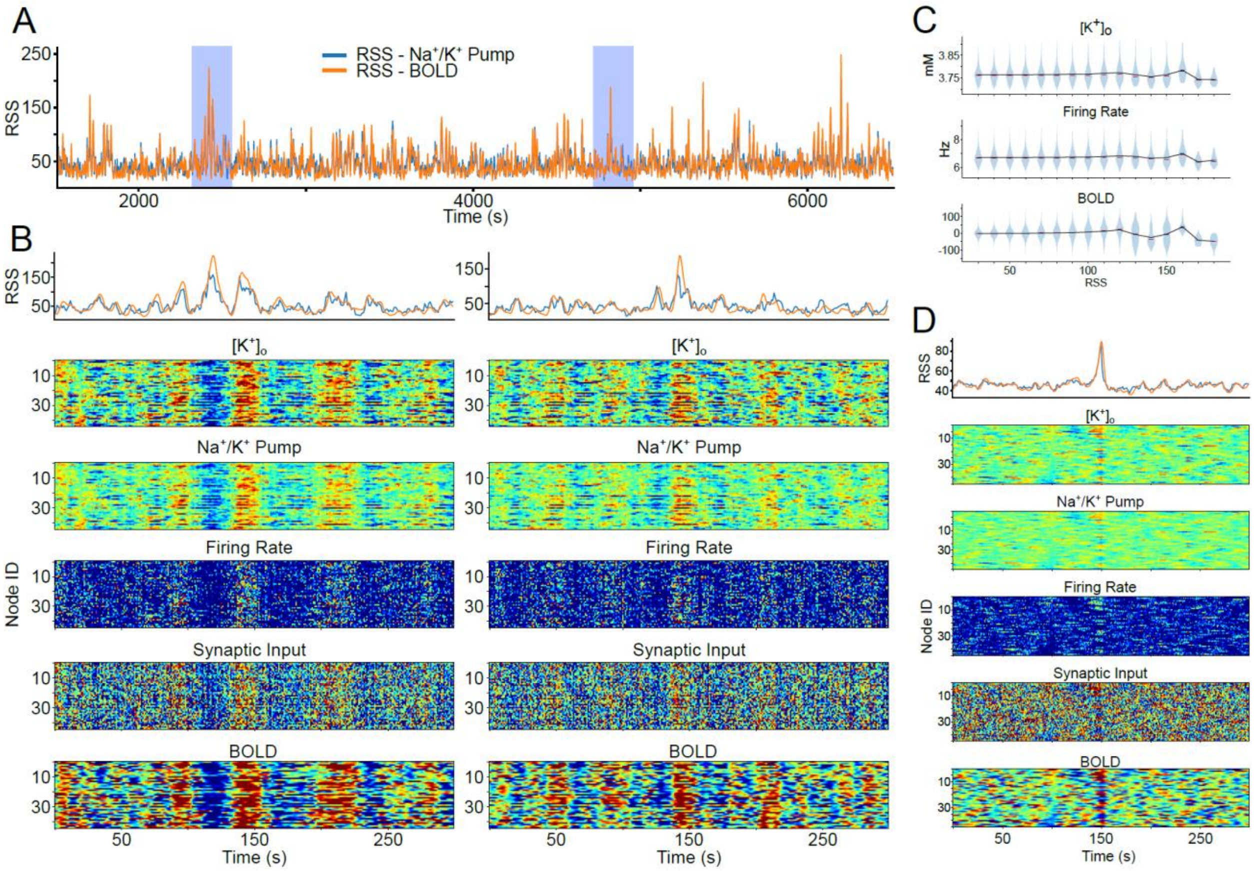

Figure S1. A. Trace of edge-based time series estimated for entire human connectome network for 1000sec simulation. B. Zoom-in of two periods when the RSS had values above 95th percentile.

bioRxiv preprint doi: https://doi.org/10.1101/2025.11.08.687387; this version posted November 9, 2025. The copyright holder for this preprint (which was not certified by peer review) is the author/funder, who has granted bioRxiv a license to display the preprint in perpetuity. It is made available under aCC-BY 4.0 International license.

From top, the plots show the RSS of the whole network, extracellular K^+, Na^+/K^+ pump, firing rate, average synaptic input and BOLD across individual regions for the selected period. C. Violin plot show the distribution of $[K^+]_o$ *(top), firing rate (middle) and BOLD(bottom) for bins of RSS value from the entire simulation period. D. RSS event (defined as RSS > 95th percentile) triggered average of various neural measures across different regions within 150 sec window of the event.*
